# Exploring the Critical Factors Limiting Polyaniline Biocompatibility

**DOI:** 10.3390/polym11020362

**Published:** 2019-02-19

**Authors:** Věra Kašpárková, Petr Humpolíček, Jaroslav Stejskal, Zdenka Capáková, Patrycja Bober, Kateřina Skopalová, Marián Lehocký

**Affiliations:** 1 Centre of Polymer Systems, Tomas Bata University in Zlin, Tr. T. Bati 5678, 760 01 Zlin, Czech Republic; vkasparkova@utb.cz (V.K.); capakova@utb.cz (Z.C.); skopalova@utb.cz (K.S.); lehocky@utb.cz (M.L.); 2 Department of Fat, Surfactant and Cosmetics Technology, Faculty of Technology, Tomas Bata University in Zlin, T.G.M. Sq. 5555, 760 01 Zlin, Czech Republic; 3 Polymer Centre, Faculty of Technology, Tomas Bata University in Zlin, T.G.M. Sq. 5555, 760 01 Zlin, Czech Republic; 4 Institute of Macromolecular Chemistry, Czech Academy of Sciences, Heyrovsky Sq. 2, 162 06 Prague 6, Czech Republic; stejskal@imc.cas.cz (J.S.); bober@imc.cas.cz (P.B.)

**Keywords:** polyaniline, monomer, oligomer, impurity profile, cytotoxicity

## Abstract

Today, the application of polyaniline in biomedicine is widely discussed. However, information about impurities released from polyaniline and about the cytotoxicity of its precursors aniline, aniline hydrochloride, and ammonium persulfate are scarce. Therefore, cytotoxicity thresholds for the individual precursors and their combinations were determined (MTT assay) and the type of cell death caused by exposition to the precursors was identified using flow-cytometry. Tests on fibroblasts revealed higher cytotoxicity of ammonium persulfate than aniline hydrochloride. Thanks to the synergic effect, both monomers in combination enhanced their cytotoxicities compared with individual substances. Thereafter, cytotoxicity of polyaniline doped with different acids (sulfuric, nitric, phosphoric, hydrochloric, and methanesulfonic) was determined and correlated with impurities present in respective sample (HPLC). The lowest cytotoxicity showed polyaniline doped with phosphoric acid (followed by sulfuric, methanesulfonic, and nitric acid). Cytotoxicity of polyaniline was mainly attributed to the presence of residual ammonium persulfate and low-molecular-weight polar substances. This is crucial information with respect to the purification of polyaniline and production of its cytocompatible form.

## 1. Introduction

The number of studies dealing with biomedical applications of conducting polymers in general, and polyaniline in particular, notably increased during the last decade. Thus, an understanding of the cytotoxicity of polyaniline, including the cytotoxicity of impurities leaching from the polymer, is crucial for its practical application. Polyaniline can be prepared in various forms, each differing in their chemical and physical properties. The three fundamental forms of the polymer can be synthetized [1]. At first, the green emeraldine salt is produced directly by the oxidative polymerization. During the oxidation, the completely oxidized form, blue pernigraniline salt, is observed as an intermediate formed in the presence of excess oxidant. After the oxidant is consumed, it converts to the emeraldine. If placed in alkaline media, conducting emeraldine salt converts to a nonconducting emeraldine base. Of the mentioned polyaniline forms, green polyaniline salt is of particular interest with respect to medical applications. Within the biomedical field, this conducting polymer can be used, for example, in scaffolds for bone regeneration [2], tissue engineering [3], regenerative medicine [4], or biosensing [5,6]. With regard to morphology, polyaniline can be prepared in different forms as well [7]. Here, globules, nanofibers [8,9], and nanotubes [10] can be mentioned. The preparation of polyaniline salt using chemical oxidation involves the oxidative polymerization of aniline (An) with ammonium persulfate (APS) in an acidic aqueous medium. Most frequently, syntheses are carried out in dilute hydrochloric acid or with aniline hydrochloride (AnH) as a monomer [11]. However, polyaniline can be also prepared in the presence of other inorganic and organic acids, such as sulfuric, phosphoric, nitric, and methanesulfonic acid [12]. In order to obtain the non-conducting polyaniline base, this polymerization step is followed by deprotonation of the formed polyaniline salt (usually hydrochloride) with ammonium hydroxide. The reaction course implies that substances, which can leach out of the polymer and cause polyaniline cytotoxicity, can cover residual monomers or reaction byproducts of which monomers and lower oligomers are the most probable candidates. 

The cytotoxicity and cellular response of aniline oligomers was the subject of study by Zhang et al. [13]. The results suggest that aniline trimer showed the highest cytotoxicity to the used cells, namely, mouse embryo fibroblast and human adenocarcinoma alveolar basal epithelial cells. They also demonstrated that cellular responses of aniline oligomers were influenced both by their properties and cell types used in the study. The impact of aniline oligomers on microorganisms was investigated by Gizdavic-Nikolaidis et al. [14]. It was already mentioned that polyaniline is mostly prepared using AnH (or An) and APS as the monomers/precursors. The reactants used in synthesis were widely studied in the context of their impact on human health. With respect to this issue, the following scientific works can be listed: The study on acute toxicity of AnH was published by Jenkins et al. [15] and the corresponding study dealing with APS presented by Signorin et al. [16]. Jenkins et al. [15] investigated oral administration of An in human volunteers and in rats. In general, humans were more sensitive towards action of this substance than rats. The authors found out that intake of single oral dose up to concentration of 15 mg per kg of body weight was free of any negative effect. However, concentrations above 25 mg per kg of body weight significantly increased the level of methaemoglobin in blood. Signorin et al. [16] studied the sub-chronic inhalation toxicity of APS on rats. They determined the no-observed adverse effect level (NOAEL) at 10.3 mg m^−3^ and the no-observed effect level (NOEL) at concentration of 5 mg m^−3^. In the study published by Kim et al. [17], the acute and sub-chronic oral toxicities of APS in rats were determined, and the NOAEL was set above 80 mg kg^−1^. Data on chronic toxicity of AnH were reported in U.S. EPA. Report on the safety assessment of APS used as oxidizing agent in hair bleaches and colorants provided by Pang and Fiume [18] can also be mentioned in this context. It was concluded that using of APS in this fields is safe. As can be seen, the testing of each of the substances has been conducted and the results can be found in scientific literature. However, for the practical application of polyaniline in biomedicine, it is also important to know the combined cytotoxic effect of both precursors, as they can simultaneously leach from the polymer and negatively influence its biocompatibility. Moreover, both precursors are water soluble and their residues can, therefore, be easily released from otherwise insoluble polyaniline when subjected to the physiological conditions of the body. Nevertheless, information about this combined cytotoxic effect has not yet been provided in literature and is first presented in this paper. 

The cytotoxic thresholds determined for individual polyaniline precursors and their combinations were verified in practice by means of the cytotoxicity testing of polyaniline, emeraldine salts doped with different acids, namely, hydrochloric, phosphoric, methanesulfonic, nitric, and sulfuric acids. The intention of using various dopants was to modify the polymer with different counter-ions, some of which might be less harmful in biological systems compared to the chloride anion, originating from the most frequently used precursor, aniline hydrochloride. The cytotoxicity was further related to the amount of residual AnH, APS, and low molecular impurities leaching from the polyaniline samples. 

## 2. Materials and Methods 

### 2.1. Materials Used for Synthesis

Aniline and aniline hydrochloride were purchased from Fluka (Buchs, Switzerland). Ammonium persulfate, sulfuric (S), nitric (N), phosphoric (P), and hydrochloric (Cl) acids were obtained from Lach-Ner (Neratovice, Czech Republic) and were used as delivered, without further purification. Methanesulfonic acid (M) was supplied by Sigma-Aldrich (Prague, Czech Republic). 

### 2.2. Preparation of Polyaniline Samples

Polyaniline (PANI) samples were synthetized by the oxidation of 0.2 mol L^−1^ of aniline hydrochloride with 0.25 mol L^−1^ of ammonium persulfate in the presence of 1 mol L^−1^ aqueous solution of each of the following acids: Sulfuric, nitric, phosphoric, hydrochloric, and methanesulfonic. For the polymerization, AnH (2.59 g) was dissolved in 1 mol L^-1^ acid solution to volume of 50 mL. Correspondingly, APS (5.71 g) was also dissolved in 1 mol L^−1^ acid solution to 50 mL. The aqueous solutions of both precursors were mixed in a beaker, stirred, and left at rest to polymerize overnight at laboratory temperature of 21 ± 2 °C. The next day, each of the resulting protonated, conducting PANI salts (PANI-S, PANI-N, PANI-P, PANI-Cl, PANI-M) obtained after polymerization was collected on a filter, washed with three 100 mL portions of 0.2 mol L^−1^ solution of the respective acid, then with acetone, and dried in air at room temperature. 

### 2.3. Cytotoxicity

*Cells:* Mouse embryonic fibroblast cells (ATCC CRL-1658™; NIH/3T3) were used as the model cell line. ATCC-formulated Dulbecco’s Modified Eagle’s Medium (DMEM), catalog no. 30-2002 added bovine calf serum (concentration of 10%), and penicillin/streptomycin at a concentration of 100 U mL^−1^ / 100 µg mL^−1^ (PAA Laboratories GmbH, Austria) were used as the culture medium. Cells were seeded at a concentration of 1 × 10^5^ cells mL and pre-cultivated for 24 h. The culture medium was subsequently replaced with tested extracts and monomer solutions. As a reference, cells cultivated in pure medium were used. As acidity has a negative impact on cell viability, the pH values of the tested precursor/monomer solutions (and later also PANI extracts) were verified and, if necessary, adjusted using NaHCO_3_ to physiological pH.

*Procedure:* To assess cytotoxic effects, the MTT assay (Invitrogen Corporation, USA) was performed after one-day cell cultivation at 37 ± 0.1 °C. In the test, the tetrazolium dye MTT was dissolved in ultrapure water in concentration of 5 mg mL^−1^. The culture medium was then removed and replaced by a fresh medium, and the above prepared MTT solution was added to final concentration of 0.5 mg mL^−1^. The cells were incubated with the MTT for 4 h. The formed formazan crystals were dissolved in dimethyl sulfoxide and their absorbance (corresponding to viable cells) was measured using UV-VIS spectrometry at 570 nm by an Infinite M200Pro NanoQuant (Tecan, Switzerland). All the tests were performed in quadruplicates. Dixon’s Q test was used to remove outlying values, and mean values, together with standard deviations, were calculated. Cytotoxicity was expressed as (1) mean values and standard deviations of individual absorbances; and (2) as a decrease in cell viability after exposure to the tested solutions relative to the reference. The latter procedure corresponds to that recommended by international standard EN ISO 10 993-5, in which a viability higher than 80% of the reference viability is assigned to no cytotoxicity, a viability between 60–80% to mild cytotoxicity, a viability 40–60% to moderate cytotoxicity, and, finally, a viability below 40% to severe cytotoxicity.

*Monomers and their combinations:* The cytotoxicity testing of individual precursors and their combinations was performed as follows: An, AnH, or APS were dissolved in the culture medium at concentrations ranging from 25 to 0.025 mg mL^−1^; the combinations of both precursors were tested in the following concentrations of An or AnH + APS: 5 + 0.1; 5 + 0.25; 1 + 0.25; 1 + 0.1; 0.75 + 0.5; 0.75 + 0.25; 0.75 + 0.1; 0.5 + 0.5; and 0.1 + 0.1 mg mL^−1^. The tested combinations were chosen according to the cytotoxicity threshold concentrations of the individual monomers. 

*PANI doped with different acids:* The cytotoxicity of individual polyaniline salts doped with different acids was determined using polymer extracts according to the procedure recommended in EN ISO 10993-5. Prior to testing, all samples were disinfected by dry heat at 120 °C for 40 min. The samples were extracted according to EN ISO 10993-12 in the ratio of 0.2 g of PANI powder per 1 mL culture medium in chemically inert, closed containers and at a temperature of 37 ± 1 °C under stirring for 24 ± 1 h. The parent extracts (100%) were then diluted by the culture medium to obtain solutions with concentrations of 75%, 50%, 25%, and 10% of the parent extract. All extracts were used within 24 h. Prior to testing, the pH of the extract aliquots was buffered by NaHCO_3_ to a value of approximately seven to eliminate the possible negative effect of acidic pH on cell viability.

### 2.4. Cell Death

To distinguish healthy, apoptotic, and necrotic cells after the contact of the cell cultures with selected concentrations of precursors, staining with annexin V/propidium iodide (BD Biosciences, Canada) was used. The method of cell cultivation, sample preparation, and pre-cultivation was the same as in the cytotoxicity test. Precursors were tested in concentrations 0.25 and 10 mg mL^−1^ and combinations of An+APS and AnH+APS in concentrations 0.1 + 0.1 and 5 + 0.1 mg mL^−1^. After that, the precursors solutions were removed, and the remaining adherent cells were rinsed with a phosphate buffered saline (PBS), treated with trypsin and added to a previously removed, nonadherent cell population from the same treatment. Cells were resuspended in an annexin binding buffer and stained by annexin V–FITC in a concentration of 5 µg mL^−1^ and by propidium iodide in a concentration of 5 µg mL^−1^. After 15 min in the dark, cells were analyzed by a BD FACSCanto flow cytometer (BD Biosciences, Canada).

### 2.5. Chromatographic Analysis

Analyses were conducted using an HPLC system consisting of a Waters 600E pump, a VD 040 vacuum degasser (Watrex, Prague, Czech Republic) and a UV200 ultraviolet detector (Watrex, Prague, Czech Republic). A reversed-phase C18 column X-select (300 mm × 7.8 mm; Waters) was employed. Analysis of the residual monomers and impurities was performed in an isocratic mode with a mobile phase consisting of acetonitrile/acetate buffer with pH = 4 (60/40, *v*/*v*) at a flow rate of 0.8 mL min^−1^ with a 20 µL injection volume and monitored at 235 nm by a UV detector. Data acquisition and analyses were performed with Clarity Chromatography Station. For HPLC analysis, all PANI samples were extracted in accordance with ISO 10993-12 in the ratio of 0.2 g of PANI powder per 1 mL of ultrapure water. Residual aniline, which was present in form of respective salt (aniline hydrochloride, aniline sulfate, aniline phosphate, aniline nitrate) was quantified using aniline hydrochloride standard, as all the mentioned aniline derivatives showed under chromatographic analysis identical retention time. For screening of possible impurities, other than AnH and APS present in PANI extracts, the following substances were tested: benzidine, azobenzene, hydrazobenzene, *o*-semidine, *p*-semidine, phenazine, 2-nitroaniline, 3-nitroaniline, 4-nitroaniline, *p*-aminophenol, *p*-benzoquinone, and hydroquinone (all Sigma-Aldrich, Prague, Czech Republic).

## 3. Results and Discussion

### 3.1. Cytotoxicity of Precursors

Cytotoxicity is reported according to the requirements of EN ISO 10993-5: A viability of reference is 1 and corresponds to 100% survival of cells in absence of the tested substances in cultivation medium. Values above 0.8 compared to reference are assigned to ‘no cytotoxicity’, values from 0.6 to 0.8 to ‘mild cytotoxicity’, values from 0.4 to <0.6 to ‘moderate toxicity’, and values below 0.4 to ‘severe cytotoxicity.’

**Figure 1:**
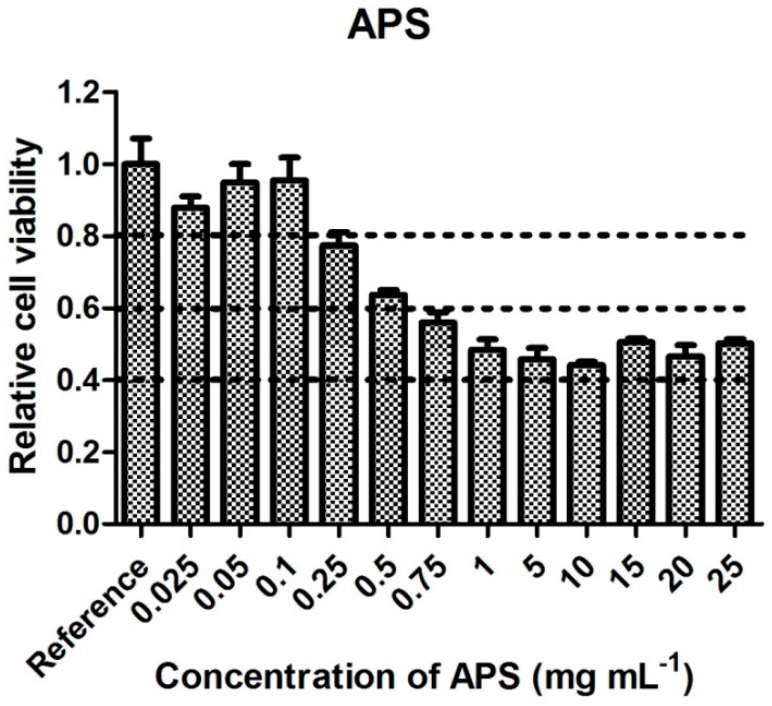
Cytotoxicity of ammonium persulfate solution.

**Figure 2:**
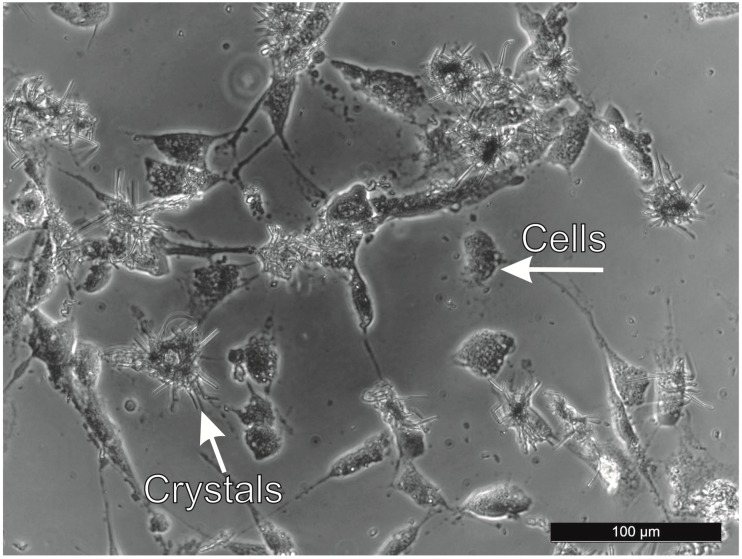
Micrograph of crystals formed after dilution of ammonium persulfate (25 mg mL^−1^) with the culture medium.

Cytotoxicity data recorded on first of the precursor APS are presented in Figure 1. From Figure 1, it is clear that APS concentrations below 0.1 mg mL^−1^ are not cytotoxic for NIH/3T3 cells, and concentrations of 0.25 and 0.5 mg mL^−1^ show only mild cytotoxicity. All other tested APS concentrations (0.75, 1, 5, 10, 15, 20, and 25 mg mL^−1^) resulted in cell viabilities ranging from 40 to 60%, indicating moderate cytotoxicity. After mixing APS at a concentration of 25 mg mL^−1^ with the culture medium, the formation of crystalline structures (Figure 2) was observed. As crystals did not form in ultrapure water at this concentration, it can be concluded that the reason for their formation is the interaction of polymer extract with some of the culture medium components. As the cultivation medium contains 0.2 g L^−1^ calcium chloride, the formation of insoluble calcium persulfate is obviously responsible for the occurrence of the crystals. 

**Figure 3:**
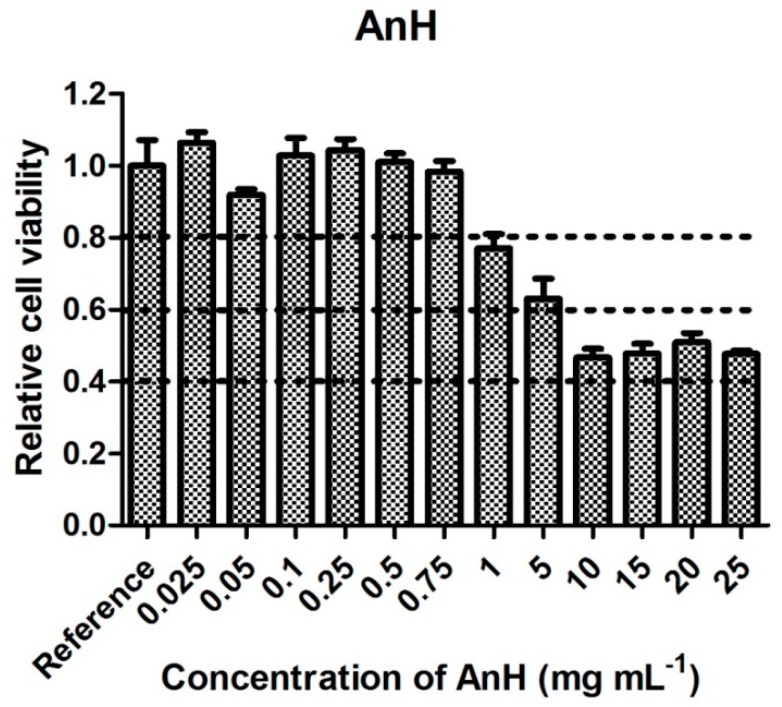
Cytotoxicity of aniline hydrochloride solution.

For the 25 mg mL^−1^ APS solution in the culture medium (the highest concentration tested), a pH of 7.3 was measured. Thanks to the buffering capacity of the medium, this pH value lies safely within the physiological range and has no negative impact on cell viability. Thus, no pH adjustment of this solution was performed. By contrast, AnH solutions, even after dilution with the medium, proved strongly acidic in nature, with pH levels of 3.9, 5.5, and 7.3 for concentrations of 25, 5, and 1 mg mL^−1^, respectively. As the buffering capacity of the medium was insufficient in this case, it was decided to adjust the pH value of the medium containing AnH to about 7 using NaHCO_3_. The cytotoxicities of the AnH solutions with adjusted pH, presented in Figure 3, indicate that solutions with concentrations of between 0.75 and 0.025 mg mL^−1^ did not influence cell viability, as 100% cell survival was detected after their application. Samples with AnH concentrations higher than 1 mg mL^−1^ exhibited mild or moderate cytotoxicity.

**Figure 4:**
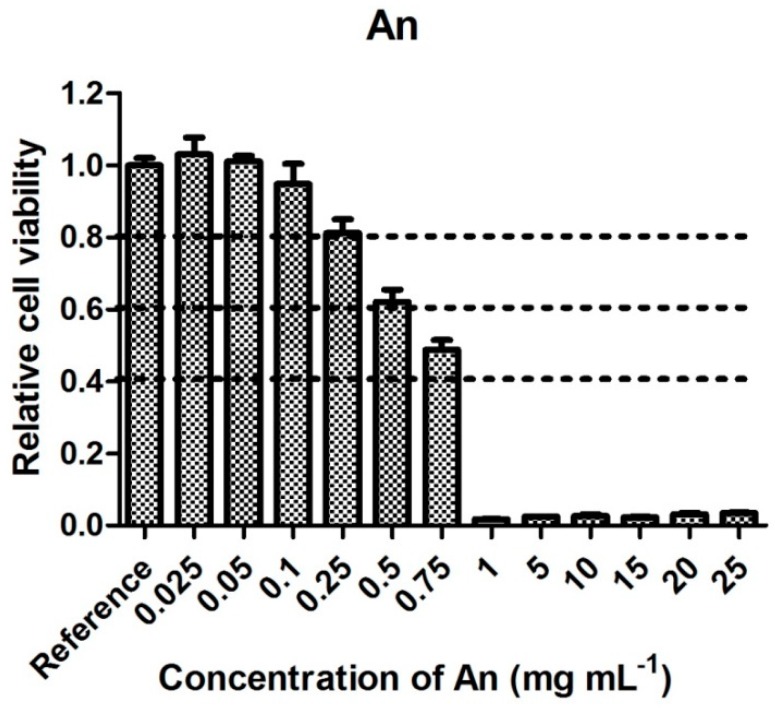
Cytotoxicity of aniline solution.

Figure 4 represents the cytotoxicities of An solutions. It is evident that An shows higher cytotoxicity than AnH. Concentrations above 1 mg mL^−1^ reach less than 5% cell viability. The concentrations without cytotoxic effect are below 0.25 mg mL^−1^.

### 3.2. Cytotoxicity of Precursors Combinations

**Figure 5:**
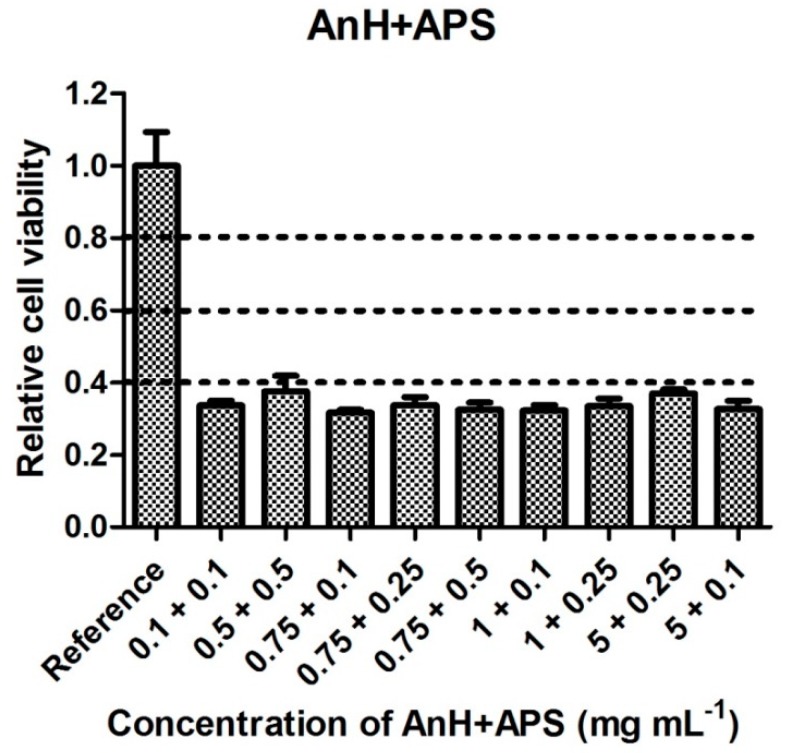
Cytotoxicity of aniline hydrochloride and ammonium persulfate in combination.

**Figure 6:**
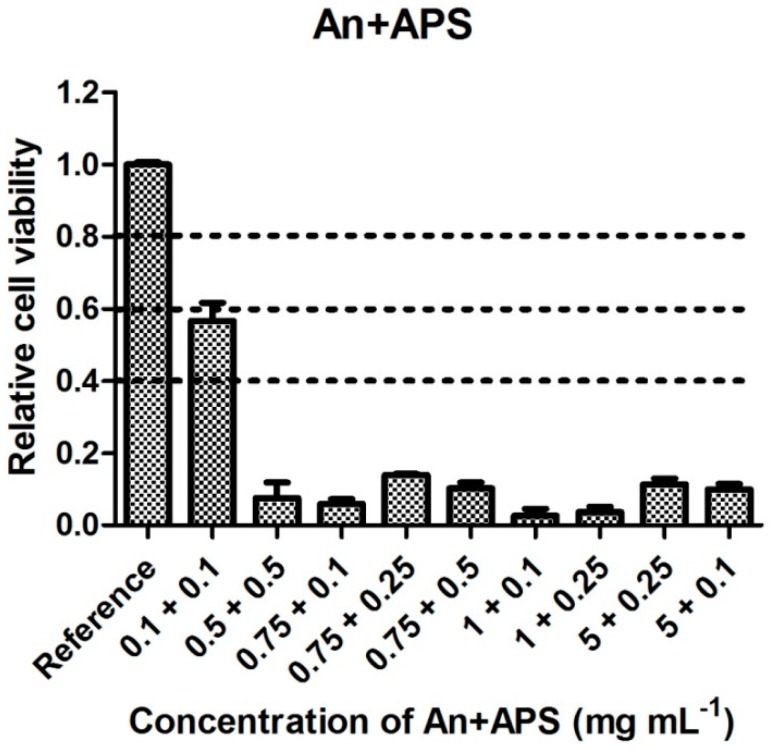
Cytotoxicity of aniline and ammonium persulfate in combination.

**Figure 7:**
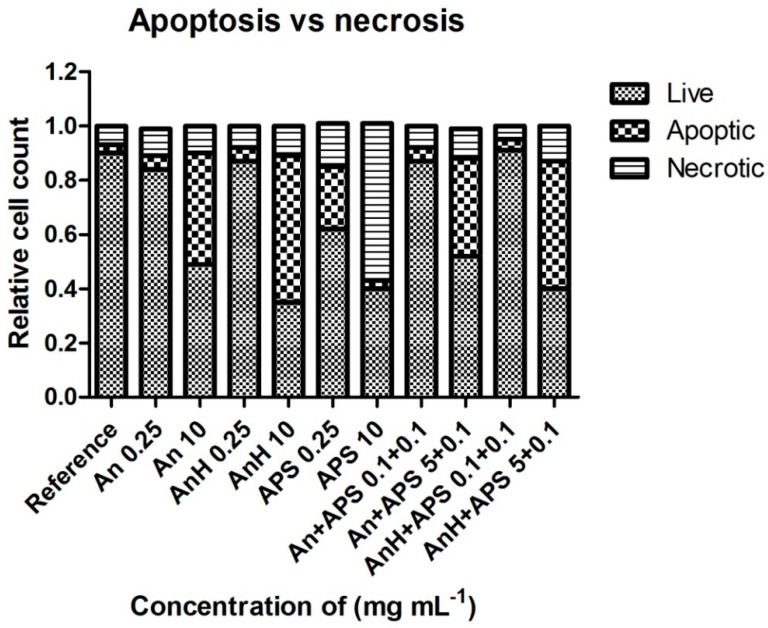
Cytotoxicity of aniline and ammonium persulfate in combination.

The cytotoxicity of precursors combined in predefined ratios is shown in Figure 5 and Figure 6. The tested concentrations were chosen according to the threshold cytotoxic concentrations recorded for each of the individual precursors. Data given in these figures clearly show the different behaviors of cells cultivated in the presence of each of the PANI precursors individually compared to their mixtures. Although the individual monomers were not cytotoxic in the respective tested concentrations, their combinations exhibited significant cytotoxicity. For example, it can be noted that neither An, AnH, nor APS, each diluted to a concentration of 0.25 mg mL^−1^, were individually cytotoxic, but after being mixed, their combination (0.1 mg mL^−1^ APS and 0.1 mg mL^−1^ AnH) exhibited high cytotoxicity with a cell survival rate of about 35%. This effect can be explained by the synergy of monomers, which, in combination, increase their harmful effects on cells. Interestingly, all the monomer mixtures used exhibited severe cytotoxicity with cell viabilities lower than 40% regardless of the An or AnH and APS concentrations used. This synergy is probably connected to different influences of each of the precursors on cell physiology, such as the effect of free radicals related to the presence of APS. It is reported that aniline derivatives possess, for example, DNA intercalating properties [19]. However, information on this effect exerted by An or AnH are insufficient. Based on the results presented in Figure 7, it can be concluded that (within the observed synergic effect) the APS plays a major role as it induces necrosis to a significantly higher extent in comparison with AnH. Its harmful effect on cell physiology or integrity of cytoplasmic membrane is probably more important than effect of An or AnH. This observation is in agreement with results published in [20], reporting on apoptotic effect and oxidative stress induced by aniline. 

### 3.3. Apoptosis vs. Necrosis Rate of Precursors and Precursors Combinations

The type of cell death caused by PANI precursors was determined using flow cytometry. From the Figure 7, it is clear that the total amount of death cells increased with the growing concentration of individual precursors. At lower concentrations of An, AnH (0.25 mg mL^−1^) and their combinations (An-APS, AnH-APS 0.1 + 0.1 mg mL^−1^) the number of healthy, apoptic, and necrotic cells was comparable to the reference. At the corresponding concentration of APS (0.25 mg mL^−1^), the amount of death cells increased, while the ratio of apoptic and necrotic cells remained similar. At the higher concentrations of precursors the quantity of death cells rapidly increased and the cells death was mostly caused by apoptosis. However, APS in concentration of 10 mg mL^−1^ induced also necrosis.

### 3.4. Cytotoxicity of Polyaniline Doped with Different Acids

Knowledge of the cytotoxicity thresholds determined for the individual precursors and their combinations served as the basis for investigation of the cytotoxicity of polyaniline doped with different acids. The motivation for the test was to evaluate whether dopant acids, other than the mostly used hydrochloride, have cytotoxic impacts on eukaryotic cells similar to those of HCl. Though the preparation and purification procedure was the same for all the studied PANI samples, it is clear that both the amounts of impurities they contained and their cytotoxicities were significantly different (Table 1).

The highest cytotoxicity was recorded for the PANI-N sample and it is worth noting that it remained almost unchanged upon dilution of the extract with the culture medium. The application of 100% and 10% extracts resulted in a level of cell viability of about 40%, which, according to ISO 10993-5, denotes severe cytotoxicity. The severe cytotoxicity of PANI-N can be attributed to the high content of unknown polar impurities (see Table 2) than to the presence of residual precursors. Cytotoxicity of PANI-M was slightly lower compared to PANI-N, with cell viability being in the range of 40–60%, which can be assigned to moderate cytotoxicity. For this sample, a surprising trend was observed showing increasing cytotoxicity of extracts with lowering of their concentrations. When 100% extract reduced cell viability to 58%, extracts with concentrations of 75% to 25% decreased cell viability to 42–47%. Upon further extract dilution, its cytotoxic action against the cells decreased again and after application of 10% extract, cell viability of 53% was determined. In contrast to the PANI-N, the cytotoxicity of PANI-M was clearly attributed to presence of APS. Changes in PANI-S cytotoxicity upon dilution showed that after the treatment with the two lowest extract concentrations, viability of cells was of about 56%, indicating moderate cytotoxicity, whereas for extracts at concentrations of 50% to 100%, cell viabilities between 60 and 80% (indicating mild cytotoxicity) were observed. The cytotoxic behavior of PANI-Cl and PANI-P upon dilution was as expected, that is, decreasing concentrations of extracts exhibited decreasing cytotoxic impacts on cells. PANI-P exhibited the lowest cytotoxicity of all tested samples, with extracts of between 100% and 50% being only mildly cytotoxic and of 25% and 10%, even showing an absence of cytotoxicity, the overall cell survival rate being more than 80%. Irrespective of the acid used, the cytotoxicities of 100% extracts were almost identical for all the tested samples (58–62% cell survival) with the exception of the PANI-N sample (41% cell survival). However, all these values are still within the category of moderate cytotoxicity. The presented results were compared with data summarized in two previously published works dealing with PANI cytotoxicity. In Humpolicek et al. [21], the cytotoxicity of polyaniline hydrochloride (PANI-H) was investigated using HaCaT cell line of human keratinocytes. This polymer should, in principle, be of the same chemical composition as PANI-Cl from the current work as both these polymers contain chloride anions originating from dopant, hydrochloric acid. However, the results show that PANI-H differs from PANI-Cl and has a similar level of cytotoxicity to PANI-M and PANI-S. When extract of PANI-H caused, at the lowest tested concentration of 10%, cell viability of 54% (moderate cytotoxicity), the corresponding extract of PANI-Cl reduced cell viability to only of 80%, which indicates mild cytotoxicity. Despite of the fact that the same dopant acid was used in PANI-Cl and PANI-H, difference between the samples can be found as regards their preparation procedure. When the samples from current work were prepared by using aniline dissolved in 1 mol L^−1^ hydrochloric acid, Humpolicek et al. [21] employed aniline hydrochloride dissolved in water, which results in 0.2 mol L^−1^ hydrochloric acid solution. This difference can lead to formation of lower content of impurities in PANI polymerized from 1 mol L^−1^ hydrochloric solution, as at higher acidity, the polymerization proceeds faster and yields a product of enhanced conductivity [11] which implies polymer with better organization of chain and lower impurity content. The remaining dopant acid used in present work, that is phosphoric had a positive impact on the cytotoxicity of PANI and PANI-P was found to perform better than the nanotubular PANI salt presented in the work of Stejskal et al. [22], the cyctotoxicity of which was the lowest ever observed on samples of polyaniline, emeraldine powder.

### 3.5. Impurities of Polyaniline Doped with Different Dopant Acids

If we consider cytotoxicity values (Figure 1, Figure 2, Figure 3, Figure 4, Figure 5 and Figure 6) and the amount of main impurities (Table 2), which leached from the tested samples containing different dopant acids, it is clear that the cytotoxicity of polyaniline extracts can mainly be attributed to the presence of APS and, in the case of PANI-N, also to unknown polar impurities. The APS possesses, namely, higher cytotoxicity than AnH and, moreover, its concentrations in extracts are significantly higher (from 9 ± 3 to 178 ± 29 mg mL^−1^) in comparison with AnH (0.07 ± 0.01 to 0.43 ± 0.06 mg mL^−1^). This most likely arises from the fact that APS is a strong oxidizing agent and generates free radicals, which can cause cell damage. Moreover, during polymerization, APS is used in excess in comparison with real stoichiometric ratio of the reaction. 

Interestingly, the cytotoxicities of individual extracts of PANI with different counter-ions did not fully correspond to impurity content of the investigated samples. According to the sum of residual APS and AnH leached from PANI after 24 h extraction in ultrapure water, the samples can be ordered as follows: PANI-S > PANI-M > PANI-Cl > PANI-N and PANI-P. This means that the highest content of these two impurities is contained in PANI-S, while the PANI-P contains their smallest amount. With regard to the real order from more to less cytotoxic, samples are in order PANI-N > PANI-M > PANI-P > PANI-S for 100% extracts, and PANI-N > PANI-M > PANI-S > PANI-Cl and PANI-P for 10% extracts. The discrepancy between content of impurities and cytotoxicity is mainly observed in case of PANI-N and this difference in behavior of PANI-N can be explained by the different impurity profile of this polymer. Compared to all other samples, this polymer contains not only residual AnH and APS, but also one unknown substance with shorter retention time, hence higher polarity, than APS. The content of this substance was of approximately 140 mg mL^−1^, as calculated according to calibration created for APS. Authors are aware of the fact that this is not the most correct way, to quantify this unknown impurity, however, under the current state of knowledge and with available HPLC detector, it was considered the best way to perform it. Here, it should be underlined that determination of the impurities was mainly focused on residual monomers and the conflict between impurity content and cytotoxicity could also be due to other impurities, for example, oligomers. In this context, the work of Zhang et al. [13] on the cellular responses of aniline oligomers is worth mentioning; Therefore, in attempt to identify this polar unknown peak observed in PANI-N, possible impurities which may arise during oxidation of AnH with APS in environment of nitric acid were considered and analyzed using HPLC. These included benzidine, azobenzene, hydrazobenzene, *o*-semidine, *p*-semidine, phenazine, 2-nitroaniline, 3-nitroaniline, 4-nitroaniline, *p*-aminophenol, *p*-benzoquinone, and hydroquinone. Unfortunately, neither of these possible impurities could be assigned to the unknown peak present in PANI-N. These analyses nevertheless confirmed that polyaniline samples do not contain cytotoxic benzidine, azobenzene or nitroanilines.

Interestingly, the cytotoxicities of polyaniline extracts also do not correspond to the cytotoxicities of monomers. According to the impurity profiles, the cytotoxicities should be higher than they really are. This is probably because a different extraction medium was used for the impurity profile (ultrapure water) than for the cytotoxicity assay (the cultivation medium). The cultivation medium contained a number of compounds and macromolecules which could interact with impurities and block/reduce their effect.

## 4. Conclusions

Information about impurities released from polyaniline prepared using different dopant acids, namely sulfuric, nitric, phosphoric, hydrochloric, and methanesulfonic is presented for the first time. Together with the determination of the cytotoxicity of precursors (An, AnH, APS), it provides substantial information needed for the purification of polyaniline and its modifications leading to preparation of noncytotoxic, biocompatible form of this polymer. Tests performed on fibroblast cell line NIH/3T3 revealed higher cytotoxicity of APS in comparison with An and AnH. Thanks to synergic effect, both monomers in combinations significantly enhanced their cytotoxicities compared with individual substances. Analyses also revealed that polyaniline doped with phosphoric acid showed the most promising properties among the remaining polymers doped with other tested acids, with absence of cytotoxicity observed at extract concentration of 25%. This finding also complied with the lowest content of impurities present in this sample determined by HPLC. Our study also clearly demonstrated that polyaniline cytotoxicity is mainly attributed to the presence of APS and low-molecular polar substances. Thus, the purification procedure or modifications to the method of preparation should be focused on APS. The results of the study extend information leading to design of noncytotoxic, biocompatible polyaniline.

## Figures and Tables

**Table 1:** Cytotoxicity of polyaniline extracts of various concentrations presented as average absorbance ± SD (Abs) and as relative value compared to reference (RV) according to ISO 10 993-5 standard ^1^.

Extract [%]	100	75	50	25	10
PANI-H ^2^	(C)	n.d.	(C)	(C)	(C)
Nanotubular PANI-H ^3^	(B)	n.d.	(B)	(B)	(B)
PANI-N	0.40 ± 0.01	0.38 ± 0.02	0.41 ± 0.02	0.35 ± 0.02	0.41 ± 0.02
PANI-M	0.58 ± 0.03	0.47 ± 0.02	0.47 ± 0.01	0.42 ± 0.02	0.53 ± 0.01
PANI-S	0.62 ± 0.03	0.70 ± 0.04	0.72 ± 0.01	0.56 ± 0.02	0.57 ± 0.02
PANI-Cl	n.d.	0.42 ± 0.01	0.50 ± 0.02	0.73 ± 0.01	0.80 ± 0.01
PANI-P	0.59 ± 0.05	0.60 ± 0.03	0.72 ± 0.04	0.91 ± 0.04	0.83 ± 0.03

^1^ Cytotoxicity reported according to the requirements of EN ISO 10993-5: A viability of reference corresponds to 100% cell survival; values above 0.8 compared to reference are assigned to ‘no cytotoxicity’ (A), values from 0.6 to 0.8 to ‘mild cytotoxicity’ (B), values from 0.4 to <0.6 to ‘moderate toxicity’ (C), and values below 0.4 to ‘severe cytotoxicity’ (D). ^2^ Results presented in Ref. [21]. ^3^ Results presented in Ref. [22].

**Table 2:** Impurities of AH and APS present in PANI extracts after 24 h treatment with ultrapure water at 37 °C determined by HPLC. Results expressed in mg per mL of extract.

Polyaniline Samples	Aniline Hydrochloride [mg mL^−1^]	Amonium Persulfate [mg mL^−1^]	Polar Unknown [mg mL^−1^]
PANI-N	0.32 ± 0.02	29 ± 7	140 ± 4
PANI-M	0.43 ± 0.06	86 ± 16	n.d.
PANI-S	0.39 ± 0.04	178 ± 29	n.d.
PANI-Cl	0.27 ± 0.04	35 ± 8	n.d.
PANI-P	0.07 ± 0.01	9 ± 3	n.d.

Note: n.d., not detected.
